# Immunogenicity of Current and New Therapies for Hemophilia A

**DOI:** 10.3390/ph15080911

**Published:** 2022-07-23

**Authors:** Alessandra N. L. Prezotti, Jéssica O. Frade-Guanaes, Gabriela G. Yamaguti-Hayakawa, Margareth C. Ozelo

**Affiliations:** 1 Department of Internal Medicine, School of Medical Sciences, University of Campinas, UNICAMP, Rua Tessália Vieira de Camargo, 126, Cidade Universitária, Campinas 13083-887, SP, Brazil; alprezotti@gmail.com (A.N.L.P.); jessica.oliveira.7@hotmail.com (J.O.F.-G.); hayakawa@unicamp.br (G.G.Y.-H.); 2 HEMOES, Hematology and Hemotherapy Center Dr. Marcos Daniel Santos, Av. Marechal Campos, 1468, Maruípe, Vitória 29047-105, ES, Brazil; 3 Hemocentro UNICAMP, University of Campinas, Rua Carlos Chagas, 480, Cidade Universitária, Campinas 13083-878, SP, Brazil

**Keywords:** hemophilia, factor VIII, blood coagulation factors, inhibitors, immunogenicity, plasma-derived factor VIII, recombinant factor VIII, extended half-life (EHL), emicizumab, anti-drug antibody (ADA)

## Abstract

Anti-drug antibody (ADA) development is a significant complication in the treatment of several conditions. For decades, the mainstay of hemophilia A treatment was the replacement of deficient coagulation factor VIII (FVIII) to restore hemostasis, control, and prevent bleeding events. Recently, new products have emerged for hemophilia A replacement therapy, including bioengineered FVIII molecules with enhanced pharmacokinetic profiles: the extended half-life (EHL) recombinant FVIII products. However, the main complication resulting from replacement treatment in hemophilia A is the development of anti-FVIII neutralizing alloantibodies, known as inhibitors, affecting approximately 25–30% of severe hemophilia A patients. Therefore, the immunogenicity of each FVIII product and the mechanisms that could help increase the tolerance to these products have become important research topics in hemophilia A. Furthermore, patients with inhibitors continue to require effective treatment for breakthrough bleedings and procedures, despite the availability of non-replacement therapy, such as emicizumab. Herein, we discuss the currently licensed treatments available for hemophilia A and the immunogenicity of new therapies, such as EHL-rFVIII products, compared to other products available.

## 1. Introduction

Hemophilia A is an inherited bleeding disorder caused by the deficiency or complete absence of clotting factor VIII (FVIII). This disorder is characterized by recurrent bleeding, mainly into muscles and joints, which can progress to debilitating arthropathy. Severe hemophilia A is defined as FVIII < 1% (i.e., <1 international unit per deciliter [IU/dl]) and can result in frequent spontaneous or excessive bleeding after injuries. In patients with moderate (FVIII 1 to 5 IU/dL) and mild (FVIII 6 to ≤ 40 IU/dL) hemophilia A, bleeding symptoms are usually associated with injuries and surgery [1].

For decades, replacement therapy, based on the administration of FVIII concentrates, has been the mainstay of hemophilia A treatment [2]. However, the last few decades have brought significant improvement to the treatment of hemophilia A. The development of recombinant FVIII (rFVIII) products not only resolved the issue of relying on human plasma source availability but also facilitated the development of new products. As an example, bioengineered FVIII molecules with enhanced pharmacokinetic profiles are currently available. Different technologies have been used to develop a new class of recombinant factor concentrates, the so-called extended half-life (EHL) rFVIII products [3,4]. Nevertheless, the development of inhibitors, anti-FVIII neutralizing alloantibodies, remains the main complication of hemophilia A replacement treatment. These antibodies inhibit the activity of FVIII and result in a lack of response to FVIII replacement therapy. The occurrence of inhibitors affects approximately 25–30% of severe hemophilia A patients during the first 50 exposure days (EDs) [5,6].

Several potential risk factors are associated with the development of inhibitors in hemophilia A patients. Among the possible alternatives to avoid the development of inhibitors, one of the most relevant topics has been the technology used to manufacture FVIII concentrates [5,7].

More recently, a new class of products has emerged as an alternative beyond the strategy to replace the deficient clotting factor. Non-replacement therapies, including emicizumab and rebalancing products, are effective prophylactic options for patients, regardless of the presence of inhibitors. These new products are user-friendly, with subcutaneous administration and weekly or monthly doses [4].

In this manuscript, we discuss the currently licensed treatments for hemophilia A and review the risk of inhibitor development according to each product reported. We further include the available information on the immunogenicity of the new therapies, such as EHL-rFVIII products and emicizumab. 

## 2. The Development of Anti-Factor VIII Neutralizing Antibodies (Inhibitors)

The development of inhibitors is the major drawback of replacement therapy in patients with hemophilia A. These anti-FVIII inhibitory antibodies are polyclonal and are generally of the immunoglobulin G (IgG)-4 subtype and are frequently associated with the IgG1 subtype [8].

Inhibitors in the plasma are quantified using the Nijmegen modification of the Bethesda assay for anti-FVIII inhibitory antibodies [9,10]. An inhibitor titer is represented as Bethesda unit per milliliter (BU/mL) and is defined as the dilution of patient samples required to achieve a 50% inactivation of FVIII in an equivalent volume of normal plasma. Patients with inhibitors with a peak titer < 5 BU/mL, which does not increase with exposure to additional factor products, are defined as presenting with low-responding inhibitors. In such cases, inhibitors can frequently be transitory, disappearing within six months. High-titer or high-responding inhibitors are defined as those with titers of 5 BU/mL or higher [2]. 

It is important to recognize that non-inhibitory anti-FVIII antibodies can also be present in patients with hemophilia A and even in healthy individuals [11,12]. For some hemophilia A patients, these non-inhibitory antibodies can influence the half-life of FVIII in circulation [13] and impact the efficacy of replacement therapy, although to a smaller magnitude. 

**Figure 1:**
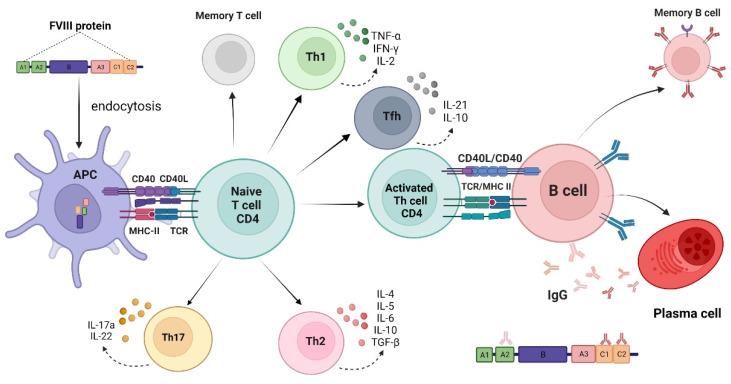
**Mechanisms of anti-FVIII alloantibody development.** Infused factor VIII (FVIII) is endocytosed by antigen-presenting cells (APC) and degraded into small peptides. FVIII-derived peptides are presented through major histocompatibility complex (MHC) class II molecules to naïve FVIII-specific CD4+ T-helper (Th) that get activated. Co-stimulatory signals are needed to elicit the immune response and release immune-regulatory molecules (cytokines). The activated T cell then interacts with B cells, which will differentiate into inhibitory antibody-secreting plasma cells. Memory T and B cells of importance for subsequent exposures are formed. Tfh, T follicular helper cells; TCR, T-cell receptor; CD40L, CD40 ligand; IL, interleukin; TNF-α, tumor necrosis factor-alpha; IFNγ, interferon-γ; TGF-β, transforming growth factor beta; IgG, immunoglobulin G.

Inhibitor development involves a complex mechanism, including central and peripheral immune tolerance, and the understanding of this mechanism may help prevent inhibitor formation. Replacement therapy with products containing FVIII protein can induce an immune response. Mechanisms of anti-FVIII alloantibody development begin with the endocytosis of the infused FVIII molecules by an antigen-presenting cell (APC) (macrophages, dendritic cells, B cells). These FVIII molecules are proteolytically degraded in peptides, defined by the major histocompatibility complex (MHC) class II molecules presented for the inactive CD4+ T-helper (Th) cell via the T-cell receptor. Co-stimulatory signals are needed to elicit the immune response and release immune-regulatory molecules (cytokines). The activated T cell then interacts with B cells, promoting differentiation and antibody formation. T follicular helper cells (Tfh) have recently been reported as a subset of CD4+ T cells that aid in the activation of B cells and are responsible for generating a T cell-dependent humoral response [14,15]. In addition, memory T and B cells of importance for subsequent exposures are formed (Figure 1).

Several genetic and non-genetic factors have been reported to be responsible for the risk of inhibitor formation. Among the genetic risk factors also recognized as unmodifiable risks, specific FVIII gene (*F8*) mutations that are responsible for the occurrence of hemophilia A, such as large deletions and nonsense mutations, are strongly correlated with inhibitor formation [16]. Additionally, a family history of inhibitors, patients of non-Caucasian ethnicity—particularly of African descent—and specific immune response genes (interleukin- [IL-] 10, cytotoxic T-lymphocyte Antigen-4 [CTLA-4], tumor necrosis factor-alpha [TNF-α]) are further responsible for a higher risk of inhibitor development [17].

The non-genetic risk factors for inhibitor development are considered potentially-modifiable risk factors. These environmental factors can be either non-related to treatment (i.e., concomitant events, including intense inflammatory response, such as surgery and infection) or treatment-related [18]. Among the treatment-related risk factors, intensity of therapy, the regimen used (on-demand or prophylaxis) [19], the source of FVIII products, which can be either plasma-derived FVIII (pdFVIII) or rFVIII products [20], and more recently modified FVIII concentrates, such as EHL-rFVIII, can be mentioned. 

## 3. Immunogenicity Aspects of Factor VIII Products

After controlling blood-borne diseases through viral inactivation techniques and the development of recombinant products, inhibitor development became the most critical safety concern for hemophilia A treatment. Therefore, the purity of pdFVIII [20] and characteristics related to the production of rFVIII [7,21] have been proposed as risk factors for inhibitor development.

Most inhibitors develop during the first 50 EDs to FVIII, particularly during the first 20 EDs [6]. Based on FVIII exposure, patients can be classified into two distinct groups: previously untreated patients (PUPs) and previously treated patients (PTPs). In order to be considered a PUP, the patient must not have received any previous treatment with factor concentrates. Nevertheless, some studies analyze PUPs and minimally-treated patients (MTPs) together. MTPs are those who were exposed to ≤4 EDs to factor concentrate or blood products. Inhibitor development is more common in PUPs, who are considered to be intolerant to exogenous FVIII. Thus, PUPs studies have become essential to determine the most relevant inhibitor risk factors, such as *F8* mutation, family history, and other genetic and non-genetic factors [18]. Despite the difficulties and limitations, PUPs studies are relevant for understanding the immunogenicity of a specific FVIII concentrate or class of FVIII products. In the United Kingdom Haemophilia Centre Doctors’ Organization (UKHCDO) Registry, the reported incidence of inhibitors among PUPs with severe hemophilia A was 64.29 per 1000 persons-year [22]. In the FranceCoag cohort, the cumulative incidence was 35% [23]. The cumulative incidence of inhibitors reported in the prospective PUPs with severe hemophilia A studies varied between 29.9–37.6% [7,24,25,26].

The definition of PTPs varies according to the study and usually includes patients who were previously exposed to FVIII concentrates for over 75 to 150 EDs [27]. However, PTPs can additionally be defined as patients tolerant to exogenous FVIII products after more than 150 EDs [6]. The reported occurrence of inhibitors in PTPs with severe hemophilia A ranges from 1.47 to 5.3 per 1000 persons-year [22,26,28,29].

Several studies reported discordant results on the immunogenicity of FVIII products. The RODIN study design was based on an extensive registry of PUPs with severe hemophilia A, the PedNet (Pediatric Network on haemophilia management). Despite having shown no significant difference in the risk of inhibitor development between pdFVIII and rFVIII users, the RODIN study showed a higher incidence of inhibitors observed in patients exposed to a second-generation full-length rFVIII produced in baby hamster kidney (BHK) cells [7]. A similar result was further observed in other cohorts of PUPs with severe hemophilia A in the UK [25] and France [23].

SIPPET (Study on Inhibitors in Plasma-Product Exposed Toddlers) is the only randomized-controlled trial designed to evaluate the immunogenicity difference between pdFVIII and rFVIII concentrates. A higher risk of inhibitors was observed among PUPs and MTPs with severe hemophilia A treated with rFVIII products (cumulative inhibitor incidence for pdFVIII and rFVIII was 26.7% and 44.5%, respectively) [5]. Some methodological issues with the SIPPET trial were heavily debated, such as the unusual geographic distribution of the study population (73% of the cohort came from India, Egypt, and Iran). Nevertheless, there are some potential explanations for the observed results. The higher immunogenicity of rFVIII could be related to different post-translational modifications, production in different cell lines, and the absence of other proteins, such as von Willebrand factor (VWF) [30].

## 4. Current Therapies for Hemophilia A

Nowadays, hemophilia A prophylaxis can be divided into replacement and non-replacement therapies. Replacement therapies comprehend both pdFVIII and rFVIII products, including standard half-life (SHL) rFVIII and EHL-rFVIII concentrates. Until 2022, emicizumab was the only licensed non-replacement therapy available.

### 4.1. Replacement Therapy: Plasma-Derived Factor VIII Concentrates

Since 1985, with the application of viral inactivation methods for manufacturing plasma-derived coagulation factor concentrates, a drastic reduction in the risk of blood-borne infections with these products has been observed [31]. Even though these products are considered safe and effective treatment alternatives, a concern of a theoretical risk of transmissible agent contamination lingered for decades. This idea boosted the perception of recombinant products as safer alternatives. However, the use of pdFVIII has regained space after the results of the SIPPET study, suggesting a lower risk of inhibitors with this type of FVIII concentrates [5].

The pdFVIII products are produced with the pooled plasma from thousands of blood donors, resulting in considerable heterogeneity in the FVIII sequence found in these products. This may result in a final FVIII product with lower immunogenicity. However, each pdFVIII concentrate can differ according to the manufacturing process used, which is mainly related to the purification method, viral inactivation process, and the protein used to stabilize and protect FVIII molecules.

In the manufacturing process, FVIII is purified from the cryoprecipitate isolated from a large volume of pooled human plasma. FVIII is finally purified via chromatographic techniques using multiple precipitations, affinity chromatography, or ion-exchange chromatography. The lyophilized FVIII then undergoes at least two methods of viral inactivation, such as heat treatment and/or solvent/detergent treatment. Some pdFVIII concentrates contain significant amounts of VWF, which is complexed with the FVIII protein, and are recognized as VWF-containing pdFVIII. Alternately, there are pdFVIII products in which albumin is added to stabilize and protect FVIII molecules. In addition, other small plasma proteins and other molecules, such as fibronectin, immunoglobulins, and fibrinogen, can be present in pdFVIII concentrates [32].

The presence of different plasma proteins may influence the immunogenicity of pdFVIII concentrates. VWF has been proposed as a critical chaperone molecule in reducing the immunogenicity of therapeutic FVIII in patients with hemophilia A. There is evidence suggesting that the inhibitor development incidence is lower when patients are treated with VWF-containing pdFVIII compared with recombinant products without VWF [33]. Other constituents that have been suggested to be involved in decreasing the immunogenicity of pdFVIII include transforming growth factor beta (TGF-β) and IL-10 [20].

Antigen competition and bystander suppression are possible mechanisms involved in the decrease of inhibitors observed among patients treated with pdFVIII products. The additional proteins present in pdFVIII concentrates may compete with FVIII for MHC class II presentation. In addition, the presence of T regulatory cells (Tregs) against these other protein components in pdFVIII may elicit bystander suppression, inhibiting FVIII-specific T cells or promoting their differentiation into Tregs [20].

Table 1 summarizes the main characteristics of pdFVIII products currently available for hemophilia A treatment.

### 4.2. Replacement Therapy: Standard Half-Life (SHL) Recombinant FVIII Products

Recombinant FVIII products became possible after cloning the FVIII gene in 1984. Less than three years later, the first clinical trials with rFVIII were initiated, and in 1992, the first rFVIII product was licensed. However, several differences can be observed in rFVIII production and formulation. One is related to the FVIII cDNA sequence used, which can be of full-length (FL) or B domain-deleted (BDD) FVIII [34].

The development of rFVIII products introduced a significant safety-related advantage, adding to the previously used viral inactivation processes in manufacturing pdFVIII: the progressive removal of animal and human proteins in these products. The different rFVIII generations are defined according to the evolution of their manufacturing process. First-generation rFVIII concentrates use animal-derived proteins in the cell culture medium, and human serum albumin is added to stabilize the final rFVIII formulation. Second-generation rFVIII concentrates use human- or animal-derived proteins in the cell culture medium, but not in the final formulation of these concentrates. Third-generation rFVIII concentrates are plasma/albumin-free products. The system used in producing these rFVIII includes a culture medium free of animal- or human-derived additives and uses different stabilizers such as sucrose, trehalose, or L- histidine [35]. We can further include the fourth-generation, which differs in the employed cell lineage. While former products use Chinese hamster ovary (CHO) or BHK cell lines, fourth-generation rFVIII is produced in the human embryonic kidney cell line (HEK293F), which ensures human-specific post-translational protein processing [36].

**Table 1:** Characteristics of plasma-derived factor VIII products currently used for hemophilia A treatment.

Product	Company	Year of First Licensing	Half-Life (Hours)	VWF:RCo/FVIII:C Ratio	Immunogenicity PTPs (%)	Immunogenicity PUPs (%)	Ref.
Hemofil M	Takeda	1966	15	NA	0	2.77	[37]
Alphanate	Grifols	1978	18	1.21	NA	NA	[38]
Humate-P	CSL Behring	1981	12.6	2.4	NA	NA	[39]
Koate-DVI	Kedrion	1992	16	1.1	NA	NA	[38]
Octanate	Octapharma	1998	11–14	0.4	0	9.8 All inhibitors 7.8 HT inhibitors	[40,41]
Wilate	Octapharma	2009	13.1 h (OSA) 11.2 h (CSA)	1.1	0	0 *	[42,43]

* Data obtained in a PUPs study, where only 11 patients were exposed to Wilate [42]. PTPs, previously treated patients; PUPs, previously untreated patients; VWF:RCo, von Willebrand ristocetin co-factor; FVIII:C, factor VIII activity; HT, high titer; OSA, one-stage clotting assay; CSA, chromogenic substrate assay; NA, not available; Ref., references.

Post-translational modifications (PTMs) can differ among rFVIII products and may influence the biosynthesis, clearance, and immunogenicity of rFVIII products. FVIII contains six sulfated tyrosine residues that play a crucial role in the FVIII activation of the coagulation pathway and the interaction between FVIII and VWF. The residue located at tyrosine 1680 (Tyr1680) is essential for interacting with VWF. Although pdFVIII is fully sulfated at Tyr1680 residue, in some rFVIII products, there is incomplete sulphation at this site, which can influence the half-life of FVIII and potentially influence the immunogenicity of this product. In addition, FVIII is a highly-glycosylated protein, and the pattern of the FVIII glycosylation differs according to the FVIII-producing cells. This could partly explain the differences in the occurrence of inhibitors among patients treated with pdFVIII and rFVIII or among rFVIII produced in different cell lines, such as second-generation rFVIII manufactured using BHK cells [20].

Differences among the SHL-rFVIII products currently available for hemophilia A treatment are shown in Table 2.

### 4.3. Replacement Therapy: Extended Half-Life (EHL) Recombinant FVIII Products

The technological evolution of the production of biosimilars, such as recombinant clotting factors, has enabled scientists to explore new alternatives. Frequent intravenous infusion is considered a significant burden in hemophilia treatment, increasing the need for central venous catheters in young patients and compromising adherence, particularly in children and adolescents. To fulfill the unmet need for a user-friendly dosing scheme, clotting factor concentrates with EHL have been developed since the 2010s [59,60].

The EHL clotting factor concentrates must have an increased half-life by at least 1.3 times over SHL products [3]. The average half-life of FVIII ranges between 10 to 14 hours, which may be lower in pediatric patients and, despite age, can additionally be influenced by body weight, VWF concentration, and ABO blood group [61,62]. Different technologies have been used to increase FVIII half-life, including conjugation with polyethylene glycol (PEG) molecule [63,64,65] and fusion with other proteins such as the fragment crystallizable (Fc) of IgG1 [66]. However, the new bioengineered molecules of FVIII have achieved a minor improvement in the half-life—only 1.5 to 1.8 times compared to SHL-rFVIII products. This modest improvement in FVIII pharmacokinetics (PK) parameters is probably due to the VWF half-life, which binds to FVIII in the circulation and protects FVIII from otherwise rapid clearance [60].

Recently, BIVV001 (rFVIIIFc-VWF-XTEN, efanesoctocog alfa) was developed. This new EHL-rFVIII incorporates two new technologies and comprehends a single rFVIII molecule fused to dimeric Fc, a D′D3 domain of VWF (FVIII–binding domain), and two XTEN hydrophilic polypeptides. The covalent link to the VWF D′D3 domain prevents binding between rFVIII and endogenous VWF. BIVV001 has a half-life that is three to four times longer than that of SHL-rFVIII products [67], resulting in a clinical benefit similar to what was observed with EHL recombinant factor IX products. Until early 2022, BIVV001 remains in phase 3 clinical trial (NCT04161495 and NCT04644575) and is not yet commercially available [68].

Regarding EHL-rFVIII immunogenicity, the recently published studies did not demonstrate a higher inhibitor occurrence compared to other SHL-rFVIII products (Table 3). Furthermore, additional concerns should be observed depending on the technology used, such as the possibility of anti-PEG antibody occurrence. Although free PEG molecules in solution are known to be non-immunogenic, when conjugated, as in PEGylated drugs, they can, on some occasions, induce an immune response. The presence of anti-PEG IgG and IgM can cause a loss of efficacy due to the acceleration of drug clearance. In addition, hypersensitivity reactions with allergic symptoms have been reported [69].

For several decades, PEG has been widely present in cosmetics, processed foods, pharmaceuticals, hygiene products, household cleaning products, agriculture, and industrial manufacturing. The reported prevalence of pre-existing anti-PEG antibodies in the general population was up to 44% [70]. In fact, in the pediatric pivotal clinical trial of turoctocog alfa pegol (N8-GP, Novo Nordisk), pathfinder™ 5, 21 severe hemophilia A patients (31.4 %) were observed to have anti-PEG antibodies before receiving N8-GP. Yet, the study reported that the presence of anti-PEG antibodies did not affect FVIII activity or the product’s efficacy [71]. In the PROTECT VIII Kids study, the pivotal pediatric clinical trial of damoctocog alfa pegol (JIVI^®^, Bayer), 11 severe hemophilia A patients (34.4%) younger than six years old discontinued the study. The reasons for the discontinuation included loss of efficacy and/or hypersensitivity reactions that occurred within the first four EDs. Four patients tested positive for the anti-PEG antibodies at screening and/or during the study [72].

The advent of EHL-rFVIII products enables more flexible treatment regimens, which are adjustable to real-world clinical use and patient lifestyle. These characteristics contribute to improving treatment individualization, with better adherence by reducing the number of infusions and/or targeting higher trough levels [73], and, therefore, have a relevant impact on treatment efficacy.

### 4.4. Non-Replacement Therapy for Hemophilia A: Emicizumab

Replacement therapy has been the standard treatment option for hemophilia for several decades. However, a significant improvement was recently achieved with the development of non-replacement therapies. This class of new agents aims to either restore the hemostasis using mimetic products or establish a unique balance of the hemostasis, thereby inhibiting the anticoagulant pathways [60].

Emicizumab is the first non-replacement agent licensed for prophylaxis to prevent or reduce the frequency of bleeding episodes in patients with hemophilia A, regardless of the presence of anti-FVIII inhibitors. Emicizumab is a humanized bispecific monoclonal antibody with binding sites to activated factor IX and factor X, mimicking the co-factorial activity of FVIII [74]. In addition, emicizumab is administered subcutaneously, dosing each 1, 2, or 4 weeks, thereby overcoming the burden associated with frequent intravenous injections, with the same effectiveness regardless of age [4,60].

Emicizumab has low immunogenicity, with anti-drug antibodies (ADAs) reported in 34 (5.1%) of 668 male hemophilia A patients with and without FVIII inhibitors, which were pooled from seven phase 3/3b clinical studies (HAVEN 1–5, HOHOEMI, and STASEY). The patients’ median age was 28 years old (range 0.3–80), including 98 patients under 12 years of age. Another fact worthy of mention is that ADAs were transient in 14/34 (41.2%) hemophilia A patients and were associated with a decreased emicizumab concentration in only 4/668 (0.6%). Of those, only one (0.1%) discontinued emicizumab due to the loss of efficacy [75].

The use of emicizumab in PUPs with severe hemophilia A remains debatable. However, there are some advantages in very young patients due to the subcutaneous route of administration, avoiding venous access burden and postponing inhibitor development in patients at high risk of developing FVIII inhibitors. Meanwhile, this medication provides effective prevention against intracranial bleeding, a relatively common bleeding in early life. However, one potential disadvantage may be the development of FVIII inhibitors in high-risk circumstances, such as during major trauma or surgery [59]. In addition, the experience of emicizumab in very young people is limited. HAVEN 7, a phase 3b clinical trial, is ongoing to evaluate the efficacy and safety in PUPs or MTPs with hemophilia A without inhibitors, including patients from birth up to ≤12 months of age (NCT04431726) [76].

**Table 3:** Characteristics of extended half-life (EHL) recombinant factor VIII products, licensed in 2022.

Product (Brand)	Company	Year of First Licensing	Technology	Cell Line	FVIII	Half-Life (Hours)	Immunogenicity PTPs (%)	Immunogenicity PUPs (%)	Ref.
Efmoroctocog alfa (Elocta, Eloctate)	Sanofi	2014	IgG1-Fc-fusion	HEK	B-domain deleted	19 (OSA) 20.9 (CSA)	No inhibitor No anaphylaxis	31.1 All inhibitors 15.6 HT inhibitors No anaphylaxis	[66,67,77,78]
Rurioctocog alfa pegol (Adynovi, Adynovate)	Takeda	2015	Random PEGylation	CHO	full-length	14.3–16 (OSA)	No inhibitor No anaphylaxis	19.2 All inhibitors	[63,73,79]
Damoctocog alfa pegol (JIVI)	Bayer	2018	Site-specific PEGylation	BHK	B-domain deleted	19 (OSA) (>12 yo) 15–16 (OSA) (<12 yo)	No inhibitor 1.5 hypersensibility 3.7 anti-PEG Ab	NA	[64,72]
Turoctocog alfa pegol (N8-GP, Esperoct)	Novo Nordisk	2019	Site-specific glycoPEGylation	CHO	B-domain truncated	15.8–19.9 (CSA) (>12 yo) 13.2–14.2 (CSA) (<12 yo)	0.6 All inhibitors 12.3 anti-PEG Ab (>12 yo) 29.4 anti-PEG Ab (<12 yo)	29.9 All inhibitors 14.9 HT inhibitors No anaphylaxis	[65,71,80]

PTPs, previously treated patients; PUPs, previously untreated patients; FVIII, factor VIII; CHO, Chinese hamster ovary cell line, BHK, baby hamster kidney cell line; HEK, human embryonic kidney; OSA, one-stage clotting assay; CSA, chromogenic substrate assay; Ab, antibody; NA, not available; Ref., references.

## 5. Conclusions

Hemophilia A treatment has improved significantly over the last few decades. However, the occurrence of FVIII inhibitors compromises the efficacy of the replacement products used for hemophilia A therapy. Several risk factors have been associated with the development of inhibitors, including the type and class of FVIII concentrates. Understanding and recognizing the immunogenicity of FVIII products has become one of the leading research interests in recent decades.

New bioengineered clotting factors, such as EHL-rFVIII products, were developed to improve prophylaxis effectiveness and ameliorate the burdensomeness of frequent intravenous administration [4]. However, immunogenicity involving EHL-rFVIII concentrates continues to be the major adverse event associated with the use of these products. More recently, emicizumab, a non-replacement therapy, represents a unique alternative for hemophilia A patients to effectively prevent bleeding, regardless of the presence of inhibitors [60,76].

New alternatives are under development to overcome the limitations resulting from the immunogenicity of replacement therapy products to increase access to safe hemophilia A treatment options [60].

## Figures and Tables

**Table 2:** Characteristics of standard half-life (SHL) recombinant factor VIII products currently used for hemophilia A treatment.

Product (Brand)	Company	Year of First Licensing	rFVIII Generation	Cell Line	Stabilizer	FVIII	Half-Life (Hours)	Immunogenicity PTPs (%)	Immunogenicity PUPs (%)	Ref.
Octocog alfa (Recombinate)	Takeda	1992	First	CHO	Human albumin	full-length	15	0.12 All inhibitors 0.06 HT inhibitors	23.9 All inhibitors 11.3 HT Inhibitors	[44,45,46]
Octocog alfa (Kogenate FS)	Bayer	1993	Second	BHK	Sucrose	full-length	11	No inhibitors	15–50.1 All inhibitors 9.8–31.6 HT inhibitor	[9,23,47]
Octocog alfa (Advate)	Takeda	2003	Third	CHO	Trehalose	full-length	9–12	0.92 All inhibitors	29.1–38 All inhibitors 12.7–26 HT inhibitors	[48,49,50]
Moroctocog alfa (Xyntha/ReFacto AF)	Pfizer	2008	Third	CHO	Sucrose	B-domain deleted	8–11	1.47 All inhibitors	33 All inhibitors 14.5 HT inhibitors	[51,52]
Turoctocog alfa (Novoeight)	Novo Nordisk	2013	Third	CHO	Sucrose	B-domain truncated	11	No inhibitors	43.1 All inhibitors 27.6 HT inhibitors	[53,54]
Simoctocog alfa (Nuwiq)	Octapharma	2015	Fourth	HEK	Sucrose/ arginine	full-length	12–17	No inhibitors	26.7 All inhibitors 16.2 HT inhibitors	[36,55]
Octogog alfa (Kovaltry)	Bayer	2016	Third	BHK	Sucrose	full-length	12.2–14.2	0.93 All inhibitors	54.8 All inhibitors 40.5 HT inhibitors *	[56,57]
Lonoctocog alfa (Afstyla)	CSL Behring	2016	Third	CHO	Sucrose/ L-histidine,	B-domain truncated single chain	14.5	No inhibitors	52 All inhibitors 26 HT inhibitors **	[58]

* Kovaltry USA Package Insert. Ongoing Leopold Kids (Part B) (NCT01311648). ** Afstyla USA Package Insert. Ongoing CSL627 UNDERSCORE 3001 (NCT02172950). PTPs, previously treated patients; PUPs, previously untreated patients; FVIII, factor VIII; rFVIII, recombinant factor VIII; HT, high titer; CHO, Chinese hamster ovary cell line, BHK, baby hamster kidney cell line; HEK, human embryonic kidney; Ref., references.

## Data Availability

Data sharing not applicable.
